# *rac*-*N*,*N*′-Bis(1-ferrocen­yleth­yl)pyridine-2,6-dicarboxamide

**DOI:** 10.1107/S160053680900422X

**Published:** 2009-02-11

**Authors:** Pattubala A. N. Reddy, Sk. Md Nasiruzzaman, Ji Eun Lee, Tae-Jeong Kim

**Affiliations:** a Department of Applied Chemistry, College of Engineering, Kyungpook National University, Daegu 702-701, South Korea; b Central Instrument Facility, Gyeongsang National University, Jinju, South Korea

## Abstract

The title compound, [Fe_2_(C_5_H_5_)_2_(C_21_H_21_N_3_O_2_)], a potential novel *N*,*N*′,*N*′′-tridentate ligand with (non-crystallographic) *C*_2_ axial symmetry, adopts a U-shaped molecular conformation.

## Related literature

For the applications of ferrocenes, see: Feng *et al.* (2008 ▶). For the use of 1,2-disubstituted planar-chiral ferrocenes in asymmetric catalysis, see: Richards & Locke (1998 ▶); Kagan & Riant (1997 ▶). For the use of chiral *C*2-symmetric bis­ferro­cenyl­amino­phosphine ligands in asymmetric catalysis, see: Cho *et al.* (1999 ▶); Song *et al.* (1999 ▶). α-Diimine ligands are known to stablize organometallic complexes (van Koten & Vrieze, 1982 ▶) and have been widely employed in a number of catalytic reactions, see: Fache *et al.* (2000 ▶).
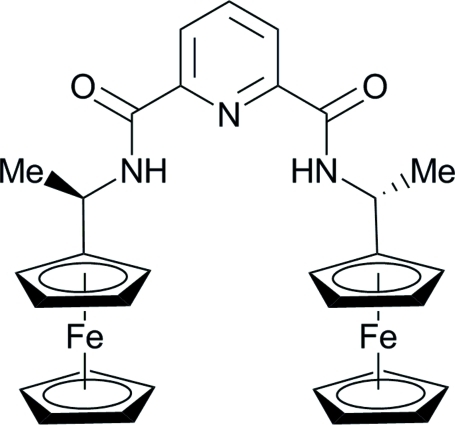


## Experimental

### 

#### Crystal data

[Fe_2_(C_5_H_5_)_2_(C_21_H_21_N_3_O_2_)]
*M* *_r_* = 589.29
Monoclinic, 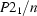

*a* = 13.1787 (8) Å
*b* = 10.2961 (6) Å
*c* = 19.8474 (12) Å
β = 103.620 (1)°
*V* = 2617.3 (3) Å^3^
*Z* = 4
Mo *K*α radiation
μ = 1.14 mm^−1^
*T* = 298 (2) K
0.41 × 0.19 × 0.15 mm

#### Data collection

Bruker SMART CCD area-detector diffractometer
Absorption correction: multi-scan (*SADABS*; Blessing, 1995 ▶; Sheldrick, 2004 ▶) *T*_min_ = 0.734, *T*_max_ = 0.842
14428 measured reflections
5126 independent reflections
3874 reflections with *I* > 2σ(*I*)
*R*_int_ = 0.046

#### Refinement

*R*[*F* ^2^ > 2σ(*F* ^2^)] = 0.098
*wR*(*F* ^2^) = 0.199
*S* = 1.20
5126 reflections
343 parameters
H-atom parameters constrained
Δρ_max_ = 0.75 e Å^−3^
Δρ_min_ = −0.61 e Å^−3^

### 

Data collection: *SMART* (Bruker, 2001 ▶); cell refinement: *SAINT* (Bruker, 2002 ▶); data reduction: *SAINT*; program(s) used to solve structure: *SHELXS97* (Sheldrick, 2008 ▶); program(s) used to refine structure: *SHELXL97* (Sheldrick, 2008 ▶); molecular graphics: *ORTEPII* (Johnson, 1976 ▶); software used to prepare material for publication: *WinGX* Publication routines (Farrugia, 1999 ▶).

## Supplementary Material

Crystal structure: contains datablocks I, global. DOI: 10.1107/S160053680900422X/tk2359sup1.cif

Structure factors: contains datablocks I. DOI: 10.1107/S160053680900422X/tk2359Isup2.hkl

Additional supplementary materials:  crystallographic information; 3D view; checkCIF report

## Figures and Tables

**Fig. 1.**
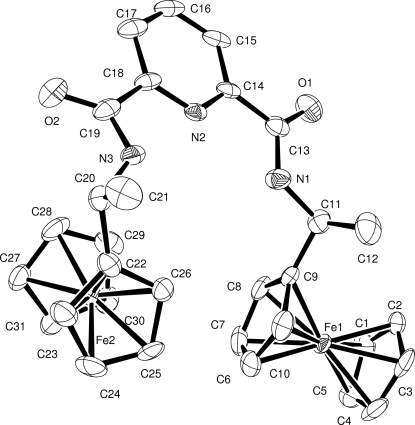
Molecular structure of (I) showing the atom numbering scheme and 30% probability thermal ellipsoids. Hydrogen atoms are omitted for clarity.

## supplementary crystallographic information

### Comment

Ferrocene derivatives have come a long way as a tool in chemistry, with applications in electrochemistry, materials science, synthesis and catalysis (Feng *et al.*, 2008). The resurgence of interest in 1,2-disubstituted planar-chiral ferrocenes has resulted in numerous interesting compounds which are finding widespread application in asymmetric catalysis (Richards & Locke, 1998; Kagan & Riant, 1997). Our past success in the use of chiral *C*2-symmetric bisferrocenyl aminophosphine ligands in asymmetric catalysis (Song *et al.*, 1999; Cho *et al.*, 1999) has prompted us to examine related bisferrocenyl analogues, such as the title complex (I), as potential sources of chiral ligand. The α-diimine ligands are now well known to stablize organometallic complexes (van Koten & Vrieze, 1982) and have thus been widely employed in a number of catalytic reactions (Fache *et al.*, 2000). Herein, an example of a completely new class of *C*2-symmetric bisferrocenyl amides, (I), that was formed *via* the reaction of 2,6-bis(chlorocarbonyl)pyridine with two equivalents of ferrocenyl ethylamine, is described.

The structure of (I), Fig. 1, shows the conformation of the nearly parallel Cp rings [the dihedral angle between their planes are 2.32 (1) and 1.04 (1)° for Fe1 and Fe2, respectively] is almost eclipsed in one ferrocene unit, whereas staggered by 5(2)° in the other. The two amide groups are nearly coplanar with the pyridine ring. The dihedral angles between the plane of the substituted Cp rings and central pyridyl ring are 86.6 (2) and 42.2 (2)°, respectively.

### Experimental

To a mixture of 2,6-bis(chlorocarbonyl)pyridine (244.0 mg, 1.21 mmol) and triethylamine (1.0 ml) in CH_2_Cl_2_ (10.0 ml) was added a solution of ferrocenyl ethylamine (0.43 g, 2.42 mmol) in CH_2_Cl_2_ (20.0 ml). The mixture was stirred at room temperature for 8 h after which it was washed with 5% HCl (3 *x* 20 ml) and 5% NaHCO_3_ (4 *x* 30 ml). The product was separated by extraction with CH_2_Cl_2_, dried over magnesium sulfate, and the solvent removed. The remaining oily residue was eluted on a silica gel column with a mixture of CH_2_Cl_2_ and MeOH (95:5). The first orange band was collected, and the solvent evaporated to give an orange solid (750 mg, 65.2% yield). Single crystals were grown by slow diffusion of hexane into a CH_2_Cl_2_ solution of (I).

### Refinement

H atoms were positioned geometrically (C—H = 0.93–0.98 Å and N—H = 0.86 Å), and refined as riding with *U*_iso_(H) = 1.2*U*_eq_(C,*N*).

### Crystal data

**Table:**

[Fe(C_5_H_5_)_2_(C_21_H_21_N_3_O_2_)]	*F*(000) = 1224
*M**_r_* = 589.29	*D*_x_ = 1.495 Mg m^−^^3^
Monoclinic, *P*2_1_/*n*	Mo *K*α radiation, λ = 0.71073 Å
Hall symbol: -P 2yn	Cell parameters from 4471 reflections
*a* = 13.1787 (8) Å	θ = 2.2–26.1°
*b* = 10.2961 (6) Å	µ = 1.14 mm^−^^1^
*c* = 19.8474 (12) Å	*T* = 298 K
β = 103.620 (1)°	Rectangular, yellow
*V* = 2617.3 (3) Å^3^	0.41 × 0.19 × 0.15 mm
*Z* = 4	

### Data collection

**Table:**

Bruker SMART CCD area-detector diffractometer	5126 independent reflections
Radiation source: fine-focus sealed tube	3874 reflections with *I* > 2σ(*I*)
graphite	*R*_int_ = 0.046
φ and ω scans	θ_max_ = 26.0°, θ_min_ = 2.1°
Absorption correction: multi-scan (*SADABS*; Blessing, 1995; Sheldrick, 2004)	*h* = −16→16
*T*_min_ = 0.734, *T*_max_ = 0.842	*k* = −12→12
14428 measured reflections	*l* = −24→17

### Refinement

**Table:**

Refinement on *F*^2^	Primary atom site location: structure-invariant direct methods
Least-squares matrix: full	Secondary atom site location: difference Fourier map
*R*[*F*^2^ > 2σ(*F*^2^)] = 0.098	Hydrogen site location: inferred from neighbouring sites
*wR*(*F*^2^) = 0.199	H-atom parameters constrained
*S* = 1.20	*w* = 1/[σ^2^(*F*_o_^2^) + (0.0634*P*)^2^ + 8.1673*P*] where *P* = (*F*_o_^2^ + 2*F*_c_^2^)/3
5126 reflections	(Δ/σ)_max_ < 0.001
343 parameters	Δρ_max_ = 0.75 e Å^−^^3^
0 restraints	Δρ_min_ = −0.61 e Å^−^^3^

### Special details

**Table:**

Experimental. Ratio of minimum to maximum apparent transmission: 0.734422
Geometry. All s.u.'s (except the s.u. in the dihedral angle between two l.s. planes) are estimated using the full covariance matrix. The cell s.u.'s are taken into account individually in the estimation of s.u.'s in distances, angles and torsion angles; correlations between s.u.'s in cell parameters are only used when they are defined by crystal symmetry. An approximate (isotropic) treatment of cell s.u.'s is used for estimating s.u.'s involving l.s. planes.
Refinement. Refinement of *F*^2^ against ALL reflections. The weighted *R*-factor *wR* and goodness of fit *S* are based on *F*^2^, conventional *R*-factors *R* are based on *F*, with *F* set to zero for negative *F*^2^. The threshold expression of *F*^2^ > 2σ(*F*^2^) is used only for calculating *R*-factors(gt) *etc*. and is not relevant to the choice of reflections for refinement. *R*-factors based on *F*^2^ are statistically about twice as large as those based on *F*, and *R*- factors based on ALL data will be even larger.

### Fractional atomic coordinates and isotropic or equivalent isotropic displacement parameters (Å^2^)

**Table:**

	*x*	*y*	*z*	*U*_iso_*/*U*_eq_	
Fe1	0.17053 (7)	0.10963 (9)	0.11043 (5)	0.0413 (3)	
Fe2	0.53645 (7)	−0.29594 (9)	0.10434 (5)	0.0417 (3)	
O1	0.4466 (5)	0.1233 (6)	0.3804 (3)	0.0812 (18)	
O2	0.8132 (4)	−0.3087 (6)	0.3000 (3)	0.0715 (16)	
N1	0.4599 (4)	0.0710 (6)	0.2728 (3)	0.0547 (16)	
H1A	0.4946	0.0250	0.2498	0.066*	
N2	0.6319 (4)	−0.0688 (5)	0.3218 (3)	0.0387 (12)	
N3	0.7391 (4)	−0.1436 (6)	0.2299 (3)	0.0457 (14)	
H3A	0.7067	−0.0710	0.2290	0.055*	
C1	0.0384 (6)	0.1732 (8)	0.1360 (4)	0.056 (2)	
H1B	0.0060	0.1346	0.1709	0.068*	
C2	0.1144 (6)	0.2705 (7)	0.1476 (4)	0.059 (2)	
H2B	0.1444	0.3116	0.1924	0.071*	
C3	0.1396 (7)	0.3010 (8)	0.0850 (5)	0.071 (2)	
H3B	0.1898	0.3667	0.0777	0.085*	
C4	0.0788 (8)	0.2198 (10)	0.0346 (4)	0.079 (3)	
H4A	0.0799	0.2183	−0.0146	0.095*	
C5	0.0169 (6)	0.1404 (9)	0.0659 (4)	0.067 (2)	
H5A	−0.0334	0.0752	0.0427	0.081*	
C6	0.2467 (6)	−0.0314 (8)	0.0700 (5)	0.068 (2)	
H6A	0.2341	−0.0524	0.0205	0.081*	
C7	0.1942 (6)	−0.0863 (7)	0.1152 (5)	0.071 (3)	
H7A	0.1404	−0.1539	0.1044	0.085*	
C8	0.2348 (5)	−0.0303 (6)	0.1797 (4)	0.0555 (19)	
H8A	0.2120	−0.0508	0.2220	0.067*	
C9	0.3101 (5)	0.0660 (6)	0.1748 (3)	0.0392 (15)	
C10	0.3179 (6)	0.0628 (8)	0.1041 (4)	0.060 (2)	
H10A	0.3644	0.1158	0.0835	0.072*	
C11	0.3724 (5)	0.1462 (7)	0.2333 (4)	0.0527 (18)	
H11A	0.3269	0.1681	0.2642	0.063*	
C12	0.4101 (7)	0.2730 (8)	0.2075 (5)	0.085 (3)	
H12A	0.4491	0.3218	0.2463	0.102*	
H12B	0.3510	0.3230	0.1836	0.102*	
H12C	0.4539	0.2540	0.1764	0.102*	
C13	0.4903 (6)	0.0677 (7)	0.3409 (4)	0.0503 (17)	
C14	0.5841 (5)	−0.0196 (6)	0.3682 (3)	0.0412 (15)	
C15	0.6128 (6)	−0.0516 (7)	0.4376 (3)	0.0530 (19)	
H15A	0.5787	−0.0141	0.4688	0.064*	
C16	0.6914 (6)	−0.1384 (8)	0.4601 (4)	0.059 (2)	
H16A	0.7109	−0.1616	0.5067	0.071*	
C17	0.7422 (6)	−0.1919 (8)	0.4129 (4)	0.058 (2)	
H17A	0.7959	−0.2519	0.4267	0.069*	
C18	0.7098 (5)	−0.1523 (7)	0.3437 (3)	0.0436 (16)	
C19	0.7603 (5)	−0.2092 (8)	0.2893 (4)	0.0521 (18)	
C20	0.7665 (5)	−0.1850 (7)	0.1659 (4)	0.0509 (18)	
H20A	0.7984	−0.2714	0.1735	0.061*	
C21	0.8435 (6)	−0.0937 (9)	0.1456 (5)	0.077 (3)	
H21A	0.9051	−0.0880	0.1826	0.092*	
H21B	0.8126	−0.0092	0.1365	0.092*	
H21C	0.8620	−0.1258	0.1046	0.092*	
C22	0.6698 (5)	−0.1939 (7)	0.1077 (3)	0.0482 (16)	
C23	0.6523 (6)	−0.2865 (8)	0.0528 (4)	0.061 (2)	
H23A	0.6994	−0.3576	0.0476	0.074*	
C24	0.5548 (7)	−0.2590 (9)	0.0074 (4)	0.070 (2)	
H24A	0.5226	−0.3076	−0.0347	0.083*	
C25	0.5118 (6)	−0.1519 (7)	0.0333 (3)	0.058 (2)	
H25A	0.4443	−0.1118	0.0124	0.069*	
C26	0.5825 (6)	−0.1105 (6)	0.0944 (3)	0.0482 (17)	
H26A	0.5719	−0.0371	0.1234	0.058*	
C27	0.5281 (6)	−0.4809 (7)	0.1373 (4)	0.063 (2)	
H27A	0.5684	−0.5549	0.1266	0.076*	
C28	0.5586 (7)	−0.3973 (8)	0.1932 (4)	0.071 (2)	
H28A	0.6241	−0.4025	0.2289	0.086*	
C29	0.4804 (7)	−0.3058 (8)	0.1908 (4)	0.069 (2)	
H29A	0.4817	−0.2353	0.2242	0.082*	
C30	0.3991 (6)	−0.3315 (8)	0.1328 (4)	0.063 (2)	
H30A	0.3341	−0.2820	0.1182	0.076*	
C31	0.4292 (6)	−0.4399 (7)	0.0982 (4)	0.066 (2)	
H31A	0.3885	−0.4802	0.0556	0.079*	

### Atomic displacement parameters (Å^2^)

**Table:**

	*U*^11^	*U*^22^	*U*^33^	*U*^12^	*U*^13^	*U*^23^
Fe1	0.0445 (5)	0.0423 (6)	0.0367 (5)	0.0193 (4)	0.0090 (4)	0.0047 (4)
Fe2	0.0507 (6)	0.0323 (5)	0.0366 (5)	0.0104 (4)	−0.0004 (4)	0.0010 (4)
O1	0.101 (5)	0.083 (4)	0.065 (4)	0.017 (4)	0.031 (3)	−0.022 (3)
O2	0.065 (4)	0.073 (4)	0.074 (4)	0.015 (3)	0.011 (3)	0.018 (3)
N1	0.043 (3)	0.075 (4)	0.044 (3)	0.014 (3)	0.006 (3)	−0.008 (3)
N2	0.036 (3)	0.040 (3)	0.036 (3)	−0.013 (2)	0.000 (2)	−0.003 (2)
N3	0.040 (3)	0.054 (4)	0.038 (3)	0.006 (3)	−0.001 (2)	−0.001 (3)
C1	0.051 (4)	0.064 (5)	0.057 (5)	0.022 (4)	0.017 (4)	−0.014 (4)
C2	0.066 (5)	0.044 (5)	0.062 (5)	0.030 (4)	0.004 (4)	−0.008 (4)
C3	0.077 (6)	0.051 (5)	0.083 (6)	0.038 (4)	0.016 (5)	0.030 (5)
C4	0.096 (7)	0.095 (7)	0.045 (5)	0.060 (6)	0.015 (5)	0.028 (5)
C5	0.050 (5)	0.076 (6)	0.064 (5)	0.033 (4)	−0.010 (4)	−0.020 (4)
C6	0.058 (5)	0.061 (5)	0.077 (6)	0.030 (4)	0.003 (4)	−0.017 (5)
C7	0.056 (5)	0.030 (4)	0.113 (8)	0.005 (3)	−0.005 (5)	−0.005 (4)
C8	0.050 (4)	0.035 (4)	0.073 (5)	0.003 (3)	−0.003 (4)	0.021 (4)
C9	0.033 (3)	0.038 (3)	0.045 (4)	0.012 (3)	0.005 (3)	0.009 (3)
C10	0.041 (4)	0.060 (5)	0.086 (6)	0.024 (4)	0.027 (4)	0.007 (4)
C11	0.041 (4)	0.056 (5)	0.061 (5)	0.008 (3)	0.012 (3)	0.000 (4)
C12	0.069 (6)	0.058 (6)	0.121 (8)	−0.014 (5)	0.006 (5)	−0.006 (5)
C13	0.052 (4)	0.050 (4)	0.047 (4)	−0.017 (3)	0.007 (3)	−0.007 (3)
C14	0.041 (4)	0.047 (4)	0.031 (3)	−0.021 (3)	−0.001 (3)	−0.005 (3)
C15	0.059 (5)	0.066 (5)	0.030 (4)	−0.029 (4)	0.002 (3)	−0.009 (3)
C16	0.061 (5)	0.071 (5)	0.034 (4)	−0.028 (4)	−0.012 (3)	0.013 (4)
C17	0.039 (4)	0.071 (5)	0.053 (4)	−0.019 (4)	−0.008 (3)	0.015 (4)
C18	0.031 (3)	0.057 (4)	0.037 (4)	−0.013 (3)	−0.003 (3)	0.009 (3)
C19	0.031 (4)	0.062 (5)	0.055 (4)	−0.006 (3)	−0.005 (3)	0.010 (4)
C20	0.050 (4)	0.054 (4)	0.050 (4)	0.012 (3)	0.013 (3)	−0.004 (3)
C21	0.057 (5)	0.104 (7)	0.077 (6)	−0.011 (5)	0.030 (4)	−0.015 (5)
C22	0.055 (4)	0.049 (4)	0.045 (4)	−0.001 (3)	0.019 (3)	−0.007 (3)
C23	0.066 (5)	0.069 (5)	0.050 (4)	0.005 (4)	0.016 (4)	−0.014 (4)
C24	0.100 (7)	0.082 (6)	0.026 (4)	−0.003 (5)	0.016 (4)	−0.010 (4)
C25	0.077 (5)	0.058 (5)	0.030 (4)	0.006 (4)	−0.005 (3)	0.021 (3)
C26	0.067 (5)	0.037 (4)	0.041 (4)	0.003 (3)	0.015 (3)	0.006 (3)
C27	0.068 (5)	0.039 (4)	0.074 (5)	0.007 (4)	−0.003 (4)	0.013 (4)
C28	0.077 (6)	0.057 (5)	0.066 (5)	−0.001 (5)	−0.013 (4)	0.034 (4)
C29	0.102 (7)	0.064 (5)	0.045 (4)	−0.022 (5)	0.028 (5)	0.003 (4)
C30	0.060 (5)	0.053 (5)	0.076 (6)	0.004 (4)	0.014 (4)	0.007 (4)
C31	0.059 (5)	0.044 (4)	0.080 (6)	−0.006 (4)	−0.013 (4)	0.005 (4)

### Geometric parameters (Å, °)

**Table:**

Fe1—C1	2.033 (7)	C8—C9	1.422 (9)
Fe1—C2	2.023 (7)	C8—H8A	0.9800
Fe1—C3	2.051 (7)	C9—C10	1.430 (10)
Fe1—C4	2.040 (7)	C9—C11	1.501 (9)
Fe1—C5	2.034 (7)	C10—H10A	0.9800
Fe1—C6	2.035 (7)	C11—C12	1.528 (11)
Fe1—C7	2.040 (7)	C11—H11A	0.9800
Fe1—C8	2.033 (6)	C12—H12A	0.9600
Fe1—C9	2.027 (6)	C12—H12B	0.9600
Fe1—C10	2.033 (7)	C12—H12C	0.9600
Fe2—C28	2.011 (7)	C13—C14	1.521 (10)
Fe2—C25	2.019 (6)	C14—C15	1.378 (9)
Fe2—C29	2.024 (7)	C15—C16	1.362 (11)
Fe2—C27	2.025 (7)	C15—H15A	0.9300
Fe2—C26	2.027 (7)	C16—C17	1.388 (11)
Fe2—C23	2.031 (8)	C16—H16A	0.9300
Fe2—C24	2.031 (7)	C17—C18	1.399 (9)
Fe2—C31	2.031 (8)	C17—H17A	0.9300
Fe2—C22	2.035 (7)	C18—C19	1.513 (10)
Fe2—C30	2.052 (8)	C20—C21	1.507 (10)
O1—C13	1.220 (8)	C20—C22	1.508 (10)
O2—C19	1.230 (8)	C20—H20A	0.9800
N1—C13	1.317 (8)	C21—H21A	0.9600
N1—C11	1.456 (9)	C21—H21B	0.9600
N1—H1A	0.8600	C21—H21C	0.9600
N2—C18	1.332 (8)	C22—C26	1.410 (9)
N2—C14	1.332 (8)	C22—C23	1.425 (9)
N3—C19	1.329 (8)	C23—C24	1.413 (11)
N3—C20	1.463 (8)	C23—H23A	0.9800
N3—H3A	0.8600	C24—C25	1.392 (11)
C1—C5	1.393 (10)	C24—H24A	0.9800
C1—C2	1.397 (11)	C25—C26	1.410 (9)
C1—H1B	0.9800	C25—H25A	0.9800
C2—C3	1.397 (11)	C26—H26A	0.9800
C2—H2B	0.9800	C27—C28	1.387 (11)
C3—C4	1.402 (13)	C27—C31	1.416 (10)
C3—H3B	0.9800	C27—H27A	0.9800
C4—C5	1.400 (12)	C28—C29	1.389 (12)
C4—H4A	0.9800	C28—H28A	0.9800
C5—H5A	0.9800	C29—C30	1.400 (11)
C6—C7	1.377 (12)	C29—H29A	0.9800
C6—C10	1.408 (11)	C30—C31	1.416 (11)
C6—H6A	0.9800	C30—H30A	0.9800
C7—C8	1.391 (11)	C31—H31A	0.9800
C7—H7A	0.9800		
			
C2—Fe1—C9	107.9 (3)	C10—C6—H6A	124.8
C2—Fe1—C1	40.3 (3)	Fe1—C6—H6A	124.8
C9—Fe1—C1	128.2 (3)	C6—C7—C8	106.6 (7)
C2—Fe1—C8	117.6 (3)	C6—C7—Fe1	70.0 (4)
C9—Fe1—C8	41.0 (3)	C8—C7—Fe1	69.8 (4)
C1—Fe1—C8	107.7 (3)	C6—C7—H7A	126.7
C2—Fe1—C10	130.8 (3)	C8—C7—H7A	126.7
C9—Fe1—C10	41.2 (3)	Fe1—C7—H7A	126.7
C1—Fe1—C10	168.0 (3)	C7—C8—C9	110.5 (7)
C8—Fe1—C10	67.8 (3)	C7—C8—Fe1	70.3 (4)
C2—Fe1—C5	67.6 (3)	C9—C8—Fe1	69.3 (4)
C9—Fe1—C5	166.1 (3)	C7—C8—H8A	124.7
C1—Fe1—C5	40.1 (3)	C9—C8—H8A	124.7
C8—Fe1—C5	128.2 (4)	Fe1—C8—H8A	124.7
C10—Fe1—C5	151.4 (3)	C8—C9—C10	105.3 (6)
C2—Fe1—C6	170.1 (4)	C8—C9—C11	126.4 (6)
C9—Fe1—C6	68.4 (3)	C10—C9—C11	128.2 (6)
C1—Fe1—C6	149.2 (4)	C8—C9—Fe1	69.7 (4)
C8—Fe1—C6	66.1 (4)	C10—C9—Fe1	69.6 (4)
C10—Fe1—C6	40.5 (3)	C11—C9—Fe1	128.0 (4)
C5—Fe1—C6	118.2 (3)	C6—C10—C9	107.1 (7)
C2—Fe1—C4	67.0 (3)	C6—C10—Fe1	69.8 (4)
C9—Fe1—C4	151.7 (4)	C9—C10—Fe1	69.2 (4)
C1—Fe1—C4	67.1 (3)	C6—C10—H10A	126.4
C8—Fe1—C4	166.8 (4)	C9—C10—H10A	126.4
C10—Fe1—C4	119.7 (4)	Fe1—C10—H10A	126.4
C5—Fe1—C4	40.2 (4)	N1—C11—C9	110.1 (6)
C6—Fe1—C4	111.6 (4)	N1—C11—C12	110.9 (6)
C2—Fe1—C7	149.0 (4)	C9—C11—C12	111.9 (6)
C9—Fe1—C7	69.2 (3)	N1—C11—H11A	107.9
C1—Fe1—C7	115.9 (4)	C9—C11—H11A	107.9
C8—Fe1—C7	39.9 (3)	C12—C11—H11A	107.9
C10—Fe1—C7	68.3 (3)	C11—C12—H12A	109.5
C5—Fe1—C7	107.5 (3)	C11—C12—H12B	109.5
C6—Fe1—C7	39.5 (3)	H12A—C12—H12B	109.5
C4—Fe1—C7	129.9 (4)	C11—C12—H12C	109.5
C2—Fe1—C3	40.1 (3)	H12A—C12—H12C	109.5
C9—Fe1—C3	117.8 (3)	H12B—C12—H12C	109.5
C1—Fe1—C3	67.7 (3)	O1—C13—N1	124.9 (7)
C8—Fe1—C3	150.9 (4)	O1—C13—C14	121.1 (7)
C10—Fe1—C3	110.5 (4)	N1—C13—C14	114.0 (6)
C5—Fe1—C3	67.9 (4)	N2—C14—C15	122.4 (7)
C6—Fe1—C3	132.6 (4)	N2—C14—C13	117.1 (6)
C4—Fe1—C3	40.1 (4)	C15—C14—C13	120.4 (7)
C7—Fe1—C3	168.8 (4)	C16—C15—C14	119.5 (7)
C28—Fe2—C25	163.7 (4)	C16—C15—H15A	120.2
C28—Fe2—C29	40.3 (3)	C14—C15—H15A	120.2
C25—Fe2—C29	126.7 (4)	C15—C16—C17	119.3 (7)
C28—Fe2—C27	40.2 (3)	C15—C16—H16A	120.3
C25—Fe2—C27	154.7 (3)	C17—C16—H16A	120.3
C29—Fe2—C27	67.7 (4)	C16—C17—C18	117.6 (7)
C28—Fe2—C26	126.1 (3)	C16—C17—H17A	121.2
C25—Fe2—C26	40.8 (3)	C18—C17—H17A	121.2
C29—Fe2—C26	107.9 (3)	N2—C18—C17	122.8 (7)
C27—Fe2—C26	163.2 (3)	N2—C18—C19	116.9 (5)
C28—Fe2—C23	119.7 (4)	C17—C18—C19	120.3 (7)
C25—Fe2—C23	68.2 (3)	O2—C19—N3	124.7 (7)
C29—Fe2—C23	153.8 (3)	O2—C19—C18	121.6 (7)
C27—Fe2—C23	108.2 (3)	N3—C19—C18	113.7 (6)
C26—Fe2—C23	68.2 (3)	N3—C20—C21	111.7 (6)
C28—Fe2—C24	154.6 (4)	N3—C20—C22	110.3 (5)
C25—Fe2—C24	40.2 (3)	C21—C20—C22	109.5 (6)
C29—Fe2—C24	164.0 (4)	N3—C20—H20A	108.4
C27—Fe2—C24	120.7 (4)	C21—C20—H20A	108.4
C26—Fe2—C24	68.1 (3)	C22—C20—H20A	108.4
C23—Fe2—C24	40.7 (3)	C20—C21—H21A	109.5
C28—Fe2—C31	68.3 (3)	C20—C21—H21B	109.5
C25—Fe2—C31	119.9 (3)	H21A—C21—H21B	109.5
C29—Fe2—C31	68.1 (4)	C20—C21—H21C	109.5
C27—Fe2—C31	40.9 (3)	H21A—C21—H21C	109.5
C26—Fe2—C31	154.3 (3)	H21B—C21—H21C	109.5
C23—Fe2—C31	126.8 (3)	C26—C22—C23	106.7 (6)
C24—Fe2—C31	108.4 (4)	C26—C22—C20	127.7 (6)
C28—Fe2—C22	107.1 (3)	C23—C22—C20	125.6 (6)
C25—Fe2—C22	68.8 (3)	C26—C22—Fe2	69.4 (4)
C29—Fe2—C22	118.9 (3)	C23—C22—Fe2	69.3 (4)
C27—Fe2—C22	125.9 (3)	C20—C22—Fe2	128.6 (5)
C26—Fe2—C22	40.6 (3)	C24—C23—C22	108.2 (7)
C23—Fe2—C22	41.0 (3)	C24—C23—Fe2	69.7 (4)
C24—Fe2—C22	68.9 (3)	C22—C23—Fe2	69.7 (4)
C31—Fe2—C22	164.1 (3)	C24—C23—H23A	125.9
C28—Fe2—C30	67.7 (3)	C22—C23—H23A	125.9
C25—Fe2—C30	108.4 (3)	Fe2—C23—H23A	125.9
C29—Fe2—C30	40.2 (3)	C25—C24—C23	108.2 (7)
C27—Fe2—C30	68.0 (3)	C25—C24—Fe2	69.4 (4)
C26—Fe2—C30	119.9 (3)	C23—C24—Fe2	69.6 (4)
C23—Fe2—C30	164.6 (3)	C25—C24—H24A	125.9
C24—Fe2—C30	127.2 (4)	C23—C24—H24A	125.9
C31—Fe2—C30	40.6 (3)	Fe2—C24—H24A	125.9
C22—Fe2—C30	153.4 (3)	C24—C25—C26	108.3 (7)
C13—N1—C11	125.3 (6)	C24—C25—Fe2	70.4 (4)
C13—N1—H1A	117.3	C26—C25—Fe2	69.9 (4)
C11—N1—H1A	117.3	C24—C25—H25A	125.9
C18—N2—C14	118.3 (6)	C26—C25—H25A	125.9
C19—N3—C20	125.4 (6)	Fe2—C25—H25A	125.9
C19—N3—H3A	117.3	C25—C26—C22	108.7 (6)
C20—N3—H3A	117.3	C25—C26—Fe2	69.3 (4)
C5—C1—C2	108.0 (8)	C22—C26—Fe2	70.0 (4)
C5—C1—Fe1	70.0 (4)	C25—C26—H26A	125.6
C2—C1—Fe1	69.5 (4)	C22—C26—H26A	125.6
C5—C1—H1B	126.0	Fe2—C26—H26A	125.6
C2—C1—H1B	126.0	C28—C27—C31	108.0 (7)
Fe1—C1—H1B	126.0	C28—C27—Fe2	69.3 (4)
C1—C2—C3	109.0 (8)	C31—C27—Fe2	69.8 (4)
C1—C2—Fe1	70.2 (4)	C28—C27—H27A	126.0
C3—C2—Fe1	71.0 (4)	C31—C27—H27A	126.0
C1—C2—H2B	125.5	Fe2—C27—H27A	126.0
C3—C2—H2B	125.5	C27—C28—C29	108.7 (7)
Fe1—C2—H2B	125.5	C27—C28—Fe2	70.5 (4)
C2—C3—C4	106.6 (8)	C29—C28—Fe2	70.4 (4)
C2—C3—Fe1	68.9 (4)	C27—C28—H28A	125.7
C4—C3—Fe1	69.5 (5)	C29—C28—H28A	125.7
C2—C3—H3B	126.7	Fe2—C28—H28A	125.7
C4—C3—H3B	126.7	C28—C29—C30	108.6 (8)
Fe1—C3—H3B	126.7	C28—C29—Fe2	69.3 (5)
C5—C4—C3	109.0 (8)	C30—C29—Fe2	71.0 (5)
C5—C4—Fe1	69.7 (4)	C28—C29—H29A	125.7
C3—C4—Fe1	70.4 (4)	C30—C29—H29A	125.7
C5—C4—H4A	125.5	Fe2—C29—H29A	125.7
C3—C4—H4A	125.5	C29—C30—C31	107.5 (7)
Fe1—C4—H4A	125.5	C29—C30—Fe2	68.8 (5)
C1—C5—C4	107.4 (8)	C31—C30—Fe2	68.9 (5)
C1—C5—Fe1	69.9 (4)	C29—C30—H30A	126.2
C4—C5—Fe1	70.1 (5)	C31—C30—H30A	126.2
C1—C5—H5A	126.3	Fe2—C30—H30A	126.2
C4—C5—H5A	126.3	C30—C31—C27	107.2 (7)
Fe1—C5—H5A	126.3	C30—C31—Fe2	70.5 (4)
C7—C6—C10	110.4 (8)	C27—C31—Fe2	69.3 (4)
C7—C6—Fe1	70.5 (5)	C30—C31—H31A	126.4
C10—C6—Fe1	69.7 (4)	C27—C31—H31A	126.4
C7—C6—H6A	124.8	Fe2—C31—H31A	126.4
